# A83 BEST PRACTICES FOR THE PROVISION OF VIRTUAL CARE IN IBD AND BEYOND: A SYSTEMATIC REVIEW OF CURRENT GUIDELINES

**DOI:** 10.1093/jcag/gwab049.082

**Published:** 2022-02-21

**Authors:** S Anvari, S Neumark, R Jangra, A Sandre, K Pasumarthi, T Xenodemetropoulos

**Affiliations:** 1 McMaster University, Hamilton, ON, Canada; 2 University of Toronto, Toronto, ON, Canada; 3 Medicine, McMaster University, Hamilton, ON, Canada

## Abstract

**Background:**

Telemedicine has emerged as a feasible adjunct to in-person care in multiple clinical contexts, including inflammatory bowel disease (IBD), and its role has expanded in the context of the COVID-19 pandemic. However, there exists a general paucity of information surrounding best practice recommendations for conducting specialty or disease-specific virtual care.

**Aims:**

The purpose of this study was to systematically review existing best practice guidelines for conducting telemedicine encounters, both in general and specific to patients with IBD.

**Methods:**

A systematic review of MEDLINE, EMBASE, and Cochrane Central Register of Controlled Trials (CENTRAL) of existing guidelines for the provision of virtual care was performed. Data was synthesized using the Synthesis Without Meta-Analysis (SWiM) guideline, and the AGREE II tool was used to evaluate quality of evidence

**Results:**

A total of 60 studies providing guidance for virtual care encounters were included; 52% of these were published during the COVID-19 pandemic. No gastroenterology-specific guidelines were found. The majority (95%) of provider guidelines specified a type of virtual encounter to which their guidelines applied. Of included studies, 65% provided guidance regarding confidentiality/security, 58% discussed technology/setup, and 56% commented on patient consent. 31 studies also provided guidance to patients or caregivers. Overall guideline quality was poor.

**Conclusions:**

General best practices for successful telemedicine encounters include ensuring confidentiality and consent, preparation prior to a visit, and clear patient communication. Future studies should aim to objectively assess the efficacy of existing clinician practices in order to further optimize the provision of virtual care for specific populations, such as patients with IBD.

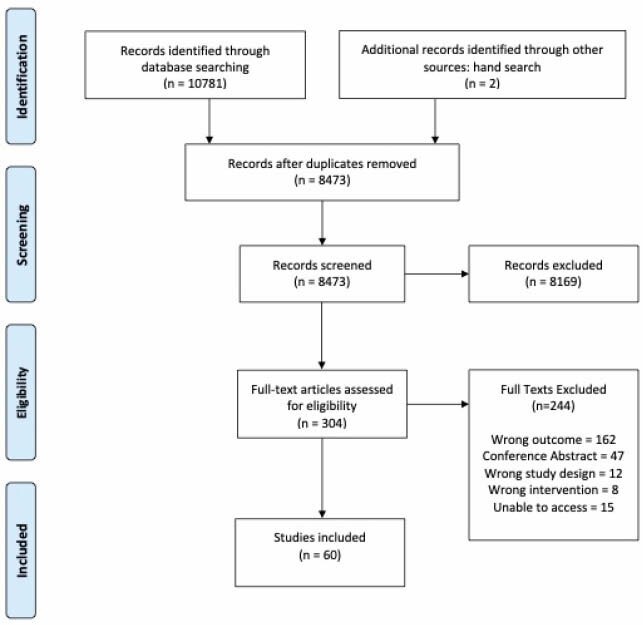

**Funding Agencies:**

None

**Table:** Summary of Best Practice Recommendations for Providers of Virtual Care

DOMAIN	RECOMMENDATIONS
Patient Selection	*Ensure that patient and case are appropriate for a telemedicine encounter
Confidentiality /Security	*Ensure encounter is being conducted in a secure environment *Communicate security of environment as well as potential for breaches of confidentiality to the patient *Identify all individuals in the room
Consent	*Ensure written consent for a telemedicine encounter is documented *Obtain verbal consent prior to beginning the encounter
Technology	*Communicate a backup plan to the patient in the event of technologic failure *Familiarize yourself with the technology prior to beginning the encounter
Physician Preparation	*Confirm technology is functioning, consent is available, and environment is ready prior to appointment *Provide patient with a checklist of any preparation prior to the visit
Competency/Training	*Ensure all staff trained in using virtual care equipment/software *Consider ongoing professional development specific to virtual care
Verbal Communication	*Speak slowly and clearly and take frequent pauses *Narrate any physical exam maneuvers
Non-Verbal Communication	*Place camera at eye level and look directly into the camera to simulate eye contact *Exaggerate non-verbal cues such as nodding and facial expressions
Documentation	*Ensure patient encounter, modality of appointment, consent, and content of visit are appropriately documented, and consider using a template
Prescribing	*Prescribe at practitioner’s discretion as would be done in an in-person visit

