# Correction to: A83 BEST PRACTICES FOR THE PROVISION OF VIRTUAL CARE IN IBD AND BEYOND: A SYSTEMATIC REVIEW OF CURRENT GUIDELINES

**DOI:** 10.1093/jcag/gwac027

**Published:** 2022-08-08

This is a correction to: S Anvari, S Neumark, R Jangra, A Sandre, K Pasumarthi, T Xenodemetropoulos, A83 BEST PRACTICES FOR THE PROVISION OF VIRTUAL CARE IN IBD AND BEYOND: A SYSTEMATIC REVIEW OF CURRENT GUIDELINES, *Journal of the Canadian Association of Gastroenterology*, Volume 5, Issue Supplement_1, March 2022, Pages 96–97, https://doi.org/10.1093/jcag/gwab049.082

In the originally published version of this manuscript the third author R Jangra’s name was incorrectly spelt “R Jhangra”.

This error has been corrected.

